# Sense of Coherence Predicts Physical Activity Maintenance and Health-Related Quality of Life: A 3-Year Longitudinal Study on Cardiovascular Patients

**DOI:** 10.3390/ijerph19084700

**Published:** 2022-04-13

**Authors:** Roberta Adorni, Andrea Greco, Marco D’Addario, Francesco Zanatta, Francesco Fattirolli, Cristina Franzelli, Alessandro Maloberti, Cristina Giannattasio, Patrizia Steca

**Affiliations:** 1 Department of Psychology, University of Milano-Bicocca, 20126 Milan, Italy; marco.daddario@unimib.it (M.D.); francesco.zanatta@unimib.it (F.Z.); patrizia.steca@unimib.it (P.S.); 2 Department of Human and Social Sciences, University of Bergamo, 24129 Bergamo, Italy; andrea.greco@unibg.it; 3 Cardiac Rehabilitation Unit, Department of Medical and Surgical Critical Care, University of Florence, 50139 Florence, Italy; francesco.fattirolli@unifi.it; 4 Azienda Ospedaliero-Universitaria Careggi, 50134 Florence, Italy; 5 Cardiac/Pulmonary Rehabilitation, ASST Gaetano Pini-CTO, 20122 Milan, Italy; cristina.franzelli@asst-pini-cto.it; 6 School of Medicine and Surgery, University of Milano-Bicocca, 20126 Milan, Italy; alessandro.maloberti@ospedaleniguarda.it (A.M.); cristina.giannattasio@unimib.it (C.G.); 7 Cardiology IV, “A. De Gasperis” Department, Ospedale Niguarda Ca’ Granda, 20162 Milan, Italy

**Keywords:** physical activity, sense of coherence, health-related quality of life, cardiovascular disease, gender, obesity

## Abstract

Cardiovascular disease (CVD) is the leading cause of morbidity and mortality globally. A physically active lifestyle can improve the health-related quality of life (HRQoL) of people with CVD. Nevertheless, adherence to a physically active lifestyle is poor. This study examined the longitudinal (pre-event, 6-, 12-, 24-, and 36-month follow-ups) physical activity profiles in 275 patients (mean age = 57.1 years; SD = 7.87; 84% men) after the first acute coronary event. Moreover, it investigated the associations among physical activity, sense of coherence (SOC), and HRQoL. Physical activity profiles were identified through latent class growth analysis, and linear regressions were then performed to explore the association between physical activity, SOC, and HRQoL. After the cardiovascular event, 62% of patients reached adequate physical activity levels and maintained them over time (virtuous profile). The remaining 38% could not implement (23%) or maintain (15%) a healthy behavior. A strong SOC at baseline (standardized β = 0.19, *p* = 0.002) predicted the probability of belonging to the virtuous profile. Moreover, a strong SOC at baseline (standardized β = 0.27, *p* < 0.001), together with the probability of belonging to the virtuous profile (standardized β = 0.16, *p* = 0.031), predicted a better HRQoL at the final follow-up. Findings showed a strong relationship between SOC, the ability to adopt a physically active lifestyle stably over time, and HRQoL in patients with CVD. They suggest the importance of tailoring physical activity interventions by promoting resilience resources such as SOC to improve patients’ quality of life after an acute coronary event.

## 1. Introduction

Cardiovascular disease (CVD) management and outcomes have improved in recent decades. Despite this, CVDs remain the leading cause of morbidity and mortality globally [1,2].

It is widely recognized that promoting a healthy lifestyle is an important way to prevent CVDs [3,4,5]. Prior research has consistently shown that encouraging physical activity can promote cardiovascular health [6]. Moreover, a physically active lifestyle is associated with a high health-related quality of life (HRQoL) in patients with coronary heart diseases [7,8,9].

International guidelines recommend that adults engage in at least 150 min per week of accumulated moderate-intensity or 75 min per week of vigorous-intensity aerobic physical activity to reduce CVD risk [1,2]. Nevertheless, despite many scientific evidence and recommendations described in the international guidelines, achieving and maintaining a physically active lifestyle remains an open challenge [6,10]. Accordingly, results from the more recent surveys conducted by the European Society of Cardiology confirmed that a large proportion of patients with CVDs do not achieve the guidelines standard for secondary prevention, including a high prevalence of physical inactivity after a cardiovascular event [11].

Recent studies focusing on the longitudinal trajectories of healthy lifestyles have highlighted that the patients with an acute coronary syndrome (ACS) after the initial adoption of a physically active lifestyle tend to drop out within six months of hospital discharge and show difficulties in maintaining adequate health-behavior patterns over time [12,13].

According to prior literature, sociodemographic and clinical factors, such as age, gender, and BMI, represent informative determinants of physical activity pattern adequateness among patients with CVDs. Specifically, being older has been associated with more sedentary behaviors and physical inactivity over time [10,14,15]. Again, prior research on patients with CVDs evidenced that men are prone to be more physically active than women [2,10,14,16,17]. Finally, lower BMI has been associated with greater adherence to a physically active lifestyle [11,18].

The role of the psychological factors in adopting and maintaining a physically active lifestyle among patients after an acute cardiovascular event is vastly underestimated.

The studies revealing an association among psychological factors, physical activity, and the ACS have focused so far on the influence of risk factors such as anxiety and depressive symptoms [12,19]. Recently, accumulating evidence has underlined the role of protective factors, suggesting that psychological resilience resources are associated with a lower risk of developing CVDs and may promote healthy behaviors and cardiovascular health [20]. Of these, sense of coherence (SOC) [21] has received remarkable attention.

SOC defines a way of thinking that enables people to identify and use the resources that are available to them: the more an individual can understand (comprehensibility), handle (manageability), and make sense of (meaningfulness) a stressful situation or disease, the greater their potential to cope with it successfully [21].

Prior research has shown that a strong SOC is associated with lower rates of CVD and mortality [22]. Moreover, previous studies on the longitudinal trajectories of HRQoL in patients with CVDs have found that compared to patients with a strong SOC, patients who had a poor or moderate SOC had a lower HRQoL over time [23,24,25]. Interestingly, previous studies have also reported associations between a strong SOC and a physically active lifestyle among the general population [26,27,28] and among patients after myocardial infarction [23,29].

Based on all these considerations, the present study explored the longitudinal (pre-event, 6-, 12-, 24-, and 36-month follow-ups) profiles of physical activity and their predictors in a cohort of 275 consecutive patients after the first acute coronary event. In addition to the relevant demographic (age, gender) and clinical (obesity) predictors, this study investigated the role of SOC. The purpose was to assess which factors could significantly predict the adherence to physicians’ prescriptions to achieve and maintain adequate physical activity. We also aimed to investigate the association between SOC, the capacity of achieving and maintaining good physical activity over time, and HRQoL.

Particularly, the first aim was to describe the longitudinal physical activity profiles over three years, following the evidence that patients with the ACS may experience different long-term physical activity trajectories [11,12].

Second, we tested the hypothesis that a strong SOC played a significant role in predicting the longitudinal profiles of physical activity, in line with previous studies showing an association between a strong SOC and a physically active lifestyle [23,26,27,28,29].

Finally, we tested the hypothesis that a strong SOC and a physically active lifestyle significantly predicted the HRQoL at the final follow-up. Indeed, prior research has shown that SOC is a strong determinant of HRQoL [23,24,30] and that a physically active lifestyle is associated with a high HRQoL [7,8] in patients with coronary heart disease. Coherently, recent evidence on patients with coronary heart disease has shown that physical inactivity and poor physical and mental HRQoL were significantly associated and suggested the need for longitudinal studies to shed light on the temporality of this association [9].

We trust that investigating the predictors of the longitudinal physical activity profiles in patients with ACS and the psychological correlates can be valuable for developing tailored cardiovascular rehabilitation programs to increase and stabilize healthy behaviors. This approach may have a significant impact on the effectiveness of healthcare practices.

## 2. Materials and Methods

### 2.1. Study Design and Participants

This study is part of a more extensive longitudinal study aimed at profiling patients with ACS and hypertension in terms of a series of behavioral, clinical, and psychological variables [12,15].

The sample consisted of 275 consecutive patients recruited between two and eight weeks after their first acute coronary event. The recruitment took place between February 2011 and October 2013 in three large public hospitals in Italy. The eligibility criteria were: age between 30 and 80 years, sufficient knowledge of the Italian language, no cognitive deficit, no comorbidity with other significant pathologies (for example, cancer). Patients were involved in cardiac rehabilitation, a medically supervised program recommended by international guidelines to patients with coronary artery disease. The hospital physicians responsible for rehabilitation recruited patients who met eligibility criteria. After recruitment, a psychologist with research experience collected information related to physical activity, SOC, and HRQoL, using the questionnaires described in the following paragraphs. Patients were evaluated at five time points (baseline, six-month, one-, two-, and three-year follow-up). During the first (baseline) assessment, the psychologist retrospectively collected information on the physical activity habitually performed before the acute coronary event. All other information collected referred to the present moment. All questionnaires were administered at each time point.

The mean age of the sample was 57.1 ± 7.87 years; 84% were men, and 16% were women. The proportion of men in the sample was a direct consequence of ACS incidence, more common among men than women [31]. All of them were Caucasian. All demographic and clinical variables are reported in Table 1 and Table 2.

The sample size adequacy was established by resorting to power analysis [32], using G*Power Version 3.1.9.7 [33]. We calculated the sample size required to perform a linear multiple regression model with the following parameters: f^2^ = 0.15 (medium effect size), α = 0.05, power = 0.95; number of predictors = 4. The sample size calculated was 129 individuals. Moreover, we computed the achieved power, given α, sample size, and effect size of the resulting model using the same software.

The Bio-Ethics Committee of all the institutions involved in the research project approved the study. Each participant provided written informed consent before their enrollment, and the entire study was conducted following the Declaration of Helsinki and all relevant guidelines and regulations covering respect for the rights and dignity of participants.

### 2.2. Measures

#### 2.2.1. Physical Activity

Physical activity was measured using the Italian version of the Rapid Assessment of Physical Activity Questionnaire (RAPA-Q) [34], a seven-item measure of the frequency and intensity of the participants’ physical activity. The questionnaire uses a yes/no scale. The total score ranges from 1 (i.e., sedentary) to 7 (i.e., regular and vigorous activity), with higher scores indicating a healthier amount of physical activity. Scores of 6 or 7 (i.e., at least 30 min of moderate to vigorous aerobic exercise five times a week) indicate the target amount of physical activity for cardiovascular prevention.

#### 2.2.2. Sense of Coherence

The Sense of Coherence Scale (SOC) [35,36] is a 13-item self-report measure of how people manage stressful situations and stay well (i.e., the sense of coherence). The SOC scale is composed of three subscales, namely comprehensibility (five items, for example: “Do you have the feeling that you are in an unfamiliar situation and do not know what to do?”), manageability (four items, for example: “How often do you have feelings that you are not sure you can keep under control?”), and meaningfulness (four items, for example: “How often do you have the feeling that there is little meaning in the things you do in your daily life?”), which can be added to a total score. Higher scores indicate higher levels of comprehensibility, manageability, and meaningfulness. All the answers were given on a 7-point Likert scale, on which the alternatives were semantically different and ranged from 1 “very seldom or never” to 7 “very often.” The scale showed a sufficient internal consistency in the present sample (Cronbach’s alpha at the baseline assessment = 0.689; it ranged from 0.612 to 0.705 in the following time points).

#### 2.2.3. Health-Related Quality of Life

Health-related quality of life was assessed with the self-report visual analog scale (VAS) of the EQ-5D, a standardized questionnaire developed by the EuroQol Group to provide a simple and generic measure of health perception for clinical appraisal [37]. The EQ-VAS records the patient’s self-rated health on a scale between 0 (worst imaginable health state) and 100 (best imaginable health state).

### 2.3. Data Analysis

Analyses were performed using the IBM SPSS Statistics, version 26 (SPSS, Chicago, IL, USA), Jamovi, version 2.2.5.0 (https://www.jamovi.org/, accessed on 23 January 2022), and Mplus software, version 7.0 [38]. All statistical tests were two-tailed, and a *p* ≤ 0.05 was considered statistically significant.

We calculated descriptive statistics on the sample’s sociodemographic, clinical, psychological, and behavioral characteristics. We reported mean and standard deviation (SD) for continuous variables and percentages for categorical variables. The data normal distribution was tested by calculating skewness and kurtosis indices, and recommended ranges of ±2 and ±7 were considered for normality, respectively [39].

Cronbach’s alpha was calculated to estimate the internal consistency of the SOC scale.

Considering both previous studies showing that SOC may change over time [24,30] and the original conceptualization by Antonovsky [35], a preliminary analysis was carried out to verify the longitudinal trajectory of the construct in the present sample. Therefore, we performed an RMANOVA, considering the total score of SOC as the dependent variable and time as the independent variable. This check made it possible to consider SOC as a stable or an evolving characteristic in the subsequent analyses.

We identified longitudinal physical activity profiles through latent class growth analysis—LCGA [40]. A number of models (from the initial model that identified one profile to the final one) were estimated by gradually increasing the profile number until the fit indices achieved the best fit. LCGA assesses the within-individual change over time by estimating an intercept (i.e., average level) and a slope (i.e., rate of change) as latent constructs [41]. We first fitted the standard LCGA with the intercept and linear effect of time. Then, we added a quadratic and cubic slope factor to allow for curved trajectories. The fit indexes included the Bayesian Information Criteria (BIC), Entropy, Lo–Mendell–Rubin Test (LMRT), and Bootstrap Likelihood Ratio Test (BLRT). Regarding BIC, smaller values indicate a better model fit. Entropy was a marker of the clarity of class delineation, with values closer to 1 indicating fewer classification errors [42]. Regarding LMRT and BLRT, a probability value <0.05 indicated that the model fit the data significantly better than the previous one. In addition, when the LMRT and BLRT values became nonsignificant (>0.05), it indicated that the previous model was the best one [43]. Generally, if a model has lower BIC, higher entropy, and achieves significant LMRT and BLRT, it has a greater fit [38].

After choosing the optimal model, we performed two multiple linear regression models. The first included the relevant sociodemographic and clinical characteristics (i.e., age, gender, obesity) and SOC measured at baseline as independent variables. The probability of belonging to the target physical activity profile was defined as the dependent variable. The second one included the SOC measured at baseline and the probability of belonging to the target physical activity profile as predictors. The HRQoL measured at the final follow-up was defined as the dependent variable. For both models, adjusted R^2^ and F test values were calculated for the explained variance and model fit, respectively.

Attrition is common in longitudinal studies [44]. We ran a Little Missing Completely At Random (MCAR) test to evaluate if data from patients who dropped at the longitudinal phase were missing at random [45]. Accordingly, parameter estimates from Mplus adjusted for missing data were determined using a robust full information maximum likelihood (FIML) estimator, which assumes data are missing at random conditional on the variables in the model [46,47,48]. FIML is a commonly accepted way to handle missing data [49,50].

## 3. Results

### 3.1. Preliminary Analysis

All continuous variables were normally distributed.

The MCAR test was not significant (χ^2^(104) = 111.4; *p* = 0.25). The nonsignificant effects of the missing data pattern (drop-outs vs. completers) suggested that all data were missing at random and that the estimates of effects were unbiased by the presence of drop-outs [51]. Thus, those who dropped out at the longitudinal phase did not significantly differ for all variables considered in this study.

As for drop-outs, 12.4% of patients were absent at the six-month follow-up. The percentage of absent patients then rose to 14.9% (12-month follow-up), 20.7% (24-month follow-up), and 33.1% (36-month follow-up). The drop-out rate at the 12-month follow-up was similar to that reported in other European studies of patients with ACS [52]. The drop-out was attributable to a relocation of the patient, their refusal to participate again in the study (no interest in participating), or, in a small minority of cases, the impossibility of tracking them. The statistical techniques of this study allowed us to use all available data and not just those provided by the completers. Therefore, the final number of participants remained equal to 275.

The RMANOVA performed considering the SOC scores as the dependent variable and time as the independent variable showed no significant effect of time (F = 0.481; *p* = 0.688), suggesting that the SOC score did not change over time. Therefore, we considered the score measured at baseline in the following analyses.

### 3.2. Longitudinal Profiles of Physical Activity

A total of five models were estimated during the exploration. The four-class solution had the lowest BIC and sufficient entropy. Moreover, the LMRT of the five-class solution was nonsignificant (Table 3). Collectively, these considerations identified the four-class solution as the optimal one.

**Figure 1:**
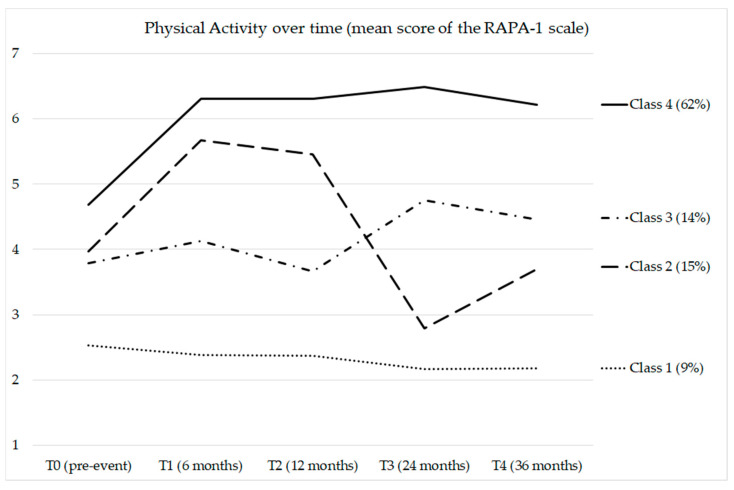
Mean scores of physical activity at each time point. A higher score reflected a higher adherence to guidelines for a healthy lifestyle.

**Figure 2:**
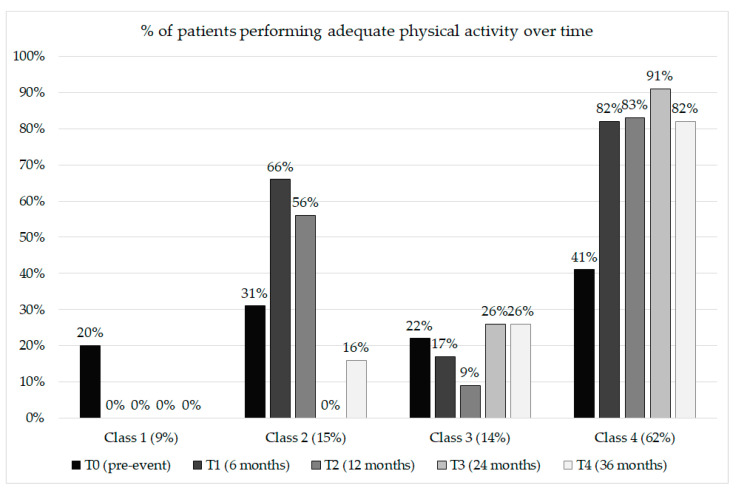
Percentages of patients performing an adequate amount of physical activity at each time point.

Table 4 reports the sociodemographic description and adequateness of physical activity for the classes identified. Figure 1 and Figure 2 illustrate the four-class solution.

Patients belonging to class 1 showed insufficient and lowered physical activity levels than patients in the other classes at T0 (only 20% achieved good physical activity before the cardiovascular event). After the cardiovascular event, the behavior worsened further. Notably, no patients achieved adequate physical activity levels during the 3-year follow-up.

Patients belonging to class 2 showed, on average, insufficient levels of physical activity before the cardiovascular event. After the cardiovascular event, the mean level of physical activity increased (66% of patients achieved good physical activity six months after the cardiovascular event). However, the patients had difficulty maintaining a healthy behavior. Indeed, at subsequent follow-ups, physical activity returned to insufficient levels, even lower than the pre-event levels.

Patients belonging to class 3 showed, on average, insufficient levels of physical activity both before and after the cardiovascular event. The cardiovascular event did not seem to change their behavior, neither improving nor worsening.

Patients belonging to class 4 showed insufficient physical activity levels but were more adequate than those from the other classes. A total of 41% achieved good physical activity before the cardiovascular event. After the cardiovascular event, most of these patients (over 80%) achieved adequate physical activity levels and maintained them over the 3-year follow-up.

### 3.3. Prediction of the Probability of Belonging to the Target Physical Activity Profile

The multiple linear regression analyses showed significant simultaneous impacts on the probability of belonging to the target physical activity profile (class 4). Gender (standardized β = 0.35, *p* = 0.033), obesity (standardized β = 0.34, *p* = 0.036), and SOC (standardized β = 0.19, *p* = 0.002) emerged as significant predictors, meaning that men, patients without the obesity risk factor, and patients with a stronger SOC were more likely to belong to the target physical activity profile. Age provided no significant impact (Table 5). The model explained the 6% of the variance and estimated a small effect size (f^2^ = 0.087; achieved power = 0.98). A significant regression equation was found (F[4, 267] = 5.62, *p* < 0.001).

### 3.4. Prediction of HRQoL at the Final Follow-Up

The multiple linear regression analyses showed significant simultaneous impacts on the HRQoL at the final follow-up (36 months). The probability of belonging to the target physical activity profile (standardized β = 0.16, *p* = 0.031) and SOC (standardized β = 0.27, *p* < 0.001) emerged as significant predictors, meaning that patients with a higher probability of belonging to the target physical activity profile and patients with a stronger SOC were more likely to report a high HRQoL at the final follow-up (Table 6). The model explained 11% of the variance and estimated a medium effect size (f^2^ = 0.14; achieved power = 0.99). Moreover, a significant regression equation was found (F[2, 167] = 11.05, *p* < 0.001).

## 4. Discussion

The present study explored the longitudinal physical activity profiles in a cohort of patients with ACS at their first event. The aim was to assess which factors could play a significant role in predicting a healthy behavior, with particular attention to the sense of coherence. Indeed, prior research evidenced an association between this psychological resilience resource and a physically active lifestyle [23,26,27,28,29]. Additionally, a physically active lifestyle and a strong SOC have been associated with improved well-being in patients with CVDs [7,8,9,23,24,25]. Therefore, this study aimed to clarify the link between these variables.

We identified four longitudinal profiles of physical activity. A fair percentage of patients (62%) after the cardiovascular event was committed to achieving adequate physical activity levels and maintaining them over time. However, a considerable percentage of patients (38%) were unable to implement or maintain a healthy behavior over time. Notably, 15% of patients in the first year after the acute cardiovascular event were committed to achieving adequate physical activity levels but tended to drop out in subsequent follow-ups. The 14% maintained levels of physical activity that were insufficient and comparable to pre-event levels; in other words, the cardiovascular event did not seem to change their behavior, neither improving nor worsening. Finally, a small percentage of patients (9%) characterized by a behavior that was, on average, worse than all the other profiles after the cardiovascular event further worsened. After the cardiovascular event, none of the patients belonging to this profile reached adequate physical activity levels.

Overall, this scenario confirms the results of the previous studies focused on the longitudinal trajectories of physical activity in patients with CVDs. A considerable proportion of patients do not achieve the guidelines standard for secondary prevention [11] or, after the initial adoption of a physically active lifestyle, tend to drop out [12,13]. Therefore, it is urgent to identify factors that may promote the achievement and maintenance of good physical activity. Our results suggest that SOC could be one of these factors.

After identifying the longitudinal profiles of physical activity, we focused on the probability of belonging to the profile that, after the cardiovascular event, reached adequate physical activity levels and maintained them over the 3-year follow-up. The aim was to test the hypotheses that being younger [10,14,15], being a man [2,10,14,16,17], being of normal weight [18], and, more interestingly, having a strong SOC [23,26,27,28,29] predicted the probability of belonging to the profile with the highest physical activity over time.

Regarding the sociodemographic variables, unexpectedly, the results did not evidence an association between age and the probability of belonging to the most virtuous physical activity profile. Previous studies suggested that being older is associated with more sedentary behaviors over time [10,14,15]. We can speculate that, in our sample, older patients could have had more free time to spend in leisure activities than the younger patients, therefore confounding the effect of age. We should also note that the age range was relatively limited and did not include the under-30 population segments.

The results showed that men were more likely than women to belong to the profile with the highest physical activity over time. This finding is consistent with previous literature. Greater physical activity was observed in men than in women in the general population [17,53], in people with cardiovascular risk factors [16], and in patients with CVD [2,10,14]. A recent systematic review [17] showed that men tend to be more physically active than women throughout the life cycle. The reasons for this gender difference appear to be related to different motivations of individuals to exercise. Men tend to report intrinsic motivations, for example, improving health, preventing noncommunicable diseases, and being competitive. On the other hand, women tend to report motivations that are more focused on social aspects, a sense of well-being, and a positive body image. Another aspect that could play a crucial role is the need of many women living in so-called high-income countries to reconcile private life and work, which may leave little room for recreational activities [53].

Results showed that patients with normal weight were more likely than patients with obesity to belong to the profile with the highest physical activity over time. This result is not surprising, as the correlation between good physical activity and reduced risk for obesity is well known [54]. Previous studies have evidenced an association between lower BMI and greater adherence over time to a physically active lifestyle in patients with CVDs [18]. A recent study also evidenced that a minority of coronary patients with obesity reported losing weight by increasing physical activity [11].

More interestingly, a strong SOC at baseline predicted the probability of belonging to the profile with the highest physical activity over time, in line with previous findings showing an association between a strong SOC and a high amount of physical activity in patients with myocardial infarction [23,29]. Our study suggests that SOC may play an important role in the active commitment to increase and maintain adequate physical activity over time. This resilience resource can stimulate change to a more physically active lifestyle in ACS patients and, most importantly, help maintain a physically active lifestyle over time. This finding is valuable considering that identifying resources to stimulate lifestyle changes is one of the essential aims of cardiac rehabilitation following an acute event. Indeed, encouraging physical activity can promote cardiovascular health [6] and well-being in patients with coronary heart diseases [7,8,9].

Finally, the results confirmed our hypothesis that a strong SOC and a physically active lifestyle significantly predicted the HRQoL at the final follow-up, coherently with previous findings showing that SOC is a strong determinant of HRQoL [23,24,30] and that a physically active lifestyle is associated with a high HRQoL [7,8,9] in patients with CVDs. Our findings enhance the current understanding of the association between physical activity, psychological resilience resources, and well-being in patients with CVDs. The positive impact of being physically active on HRQoL may have different explanations. For example, physical activity is positively associated with the ability to perform activities of daily living adequately. Accordingly, prior research has shown that a sedentary lifestyle reduces the ability to exercise and limits functional capacity, ultimately impairing both mental and physical HRQoL [9,55]. Moreover, it is possible that maintaining adequate levels of physical activity over time may have contributed to extending positive conduct to other healthy behaviors (e.g., diet, tobacco smoking, alcohol consumption), facilitating positive HRQoL outcomes. Multiple health behaviors have a concurrent effect, and simultaneously targeting them is strongly suggested [56]. Our results exclusively show the longitudinal physical activity pattern and its effects on HRQoL. However, future studies may attempt to replicate our observations by including concurrent behaviors and their prospective and simultaneous impact on well-being. Future studies may also broaden the focus to more comprehensive indicators that were not considered in this study. For example, socioeconomic status is known to significantly impact people’s physical health [57] and psychological well-being [58]. In addition, prior research has revealed that such an impact is mediated by lifestyle [59]. Following this line, future contributions may deepen the role of socioeconomic factors that may outline individual characteristics that, in turn, affect the adoption and maintenance of healthy behaviors.

The present study has some limitations. First, information on the physical activity performed prior to the acute coronary event was measured retrospectively, i.e., patients described their past habits. Thus, patients may have overestimated or underestimated the actual physical activity performed prior to the acute coronary event. Furthermore, physical activity was assessed by a self-report questionnaire and not by an objective behavioral measure. Self-report questionnaires are subjected to information bias, for example, recall bias or social desirability bias. Despite this, they are widely used in medical and psychological research and correlate with more objective measurement methods [60]. A final limitation regards the composition of our sample. On the one hand, it must be acknowledged that most of patients were men (84%). Although this composition reflects a higher incidence of ACS in men than in women known in the literature [31], this may limit the generalizability of our results to both genders. On the other hand, the homogeneity of our sample in terms of cardiovascular pathology ensures that the inferences drawn from the study apply effectively to the population of patients with ACS at their first cardiac event.

Despite limitations, this study presents valuable strengths, and its findings have important implications for behavioral interventions in patients with cardiovascular disease. The study’s most important strength was that the patients’ physical activity was evaluated over three years. Second, an advanced statistical technique (i.e., LCGA) was used to model the individual change rate from pre-event to three years after the acute coronary event.

## 5. Conclusions

This study shows that patients with ACS experience difficulties achieving and maintaining adequate levels of physical activity over time.

Our results suggest that women and patients with obesity require particular attention and should be motivated to engage in regular physical activity to promote cardiovascular health.

Moreover, our results suggest that psychological resilience resources, particularly SOC, modulate the capacity to achieve and maintain adequate physical activity and that a good physical activity, together with a strong SOC, may contribute to a better HRQoL over time.

Overall, these findings suggest the importance of effectively communicating to patients with ACS that good physical activity contributes to better cardiovascular health and improves well-being. Recognizing the benefits of well-being might stimulate lifestyle changes. Finally, our findings suggest the importance of tailoring physical activity interventions, paying particular attention to patients less prone to change their behavior (women and patients with obesity), and with the aim of promoting resilience resources such as SOC.

The topics covered in this study have become even more relevant in recent times after the pandemic outbreak. Numerous studies have investigated physical activity changes between the pre-pandemic and pandemic period, evidencing a significant decrease in physical activity and an increase in sedentary behaviors across various populations [61]. This decrease is also significant in patients with CVDs [62]. These results are not surprising: a massive reduction in exercise can be ascribed to the impossibility of leaving the house during housebound periods. Considering the relevance of a physically active lifestyle, it is of primary importance to provide people with the correct information about its role, together with guidelines to maintain and promote it even during challenging circumstances.

## Figures and Tables

**Table 1:** Sociodemographic characteristics of the sample (*n* = 275) at the baseline assessment. Means and standard deviations (SD) are reported for age. Frequencies (*n*) and percentages (%) are reported for gender, working status, educational level, and marital status.

Sociodemographic Variables	
Age, mean (SD; range)	57.1 (7.87; 34–77)
Gender, *n* (%)	
Male	231 (84%)
Female	44 (16%)
Working status, *n* (%)	
working	163 (59.3%)
not working	112 (40.7%)
Educational level, *n* (%)	
less than high school	141 (51.3%)
high school or higher	134 (48.7%)
Marital status, *n* (%)	
single\widowed\divorced	78 (28.4%)
married	197 (71.6%)

**Table 2:** Clinical characteristics of the sample (*n* = 275) at the baseline assessment. Frequencies (*n*) and percentages (%) are reported for clinical presentation, percutaneous coronary intervention, patients with at least one stent, and risk factors. Means and standard deviations (SD) are reported for body mass index, systolic blood pressure, and diastolic blood pressure.

Clinical Variables	
Clinical Presentation, *n* (%)	
Non-ST elevation myocardial infarction (NSTEMI)	54 (19.8)
ST-elevation myocardial infarction (STEMI)	196 (71.8)
Unstable Angina	23 (8.5)
Percutaneous coronary intervention, *n* (%)	258 (94.5)
Patients with at least one stent, *n* (%)	263 (96)
Risk factors, *n* (%)	
Hypertension	127 (46.5)
Dyslipidemia	143 (52.4)
Smoking History	180 (66.4)
Diabetes	47 (17.2)
Obesity	43 (15.8)
Family History of CVD	108 (39.3)
Physical Inactivity	20 (7.3)
Body Mass Index, mean (SD)	27.2 (4.1)
Systolic Blood Pressure (SBP), mean (SD)	115.9 (13.9)
Diastolic Blood Pressure (DBP), mean (SD)	72.9 (8.5)

**Table 3:** Fit indices of each model of longitudinal profiles of physical activity.

Number of Classes	BIC	E	LMRT	*p*	BLR	*p*
1	4773.11					
2	4516.77	0.77	284.43	0.249	284.43	0.000
3	4473.01	0.79	71.84	0.017	71.84	0.000
4	4447.78	0.78	53.31	0.032	53.31	0.000
5	4448.08	0.82	27.79	0.155	27.79	0.000

**Table 4:** Sociodemographic description and adequateness of physical activity for the classes identified.

				Physical Activity: Mean Score (SD) and % of Adequateness				
Class	*n* (%)	Mean Age at t0 (SD)	*n* Men (%)	t0	t1	t2	t3	t4
1	25 (9)	56.9 (7.9)	20 (80)	2.53 (1.84) 20%	2.38 (1.20) 0%	2.37 (0.99) 0%	2.17 (0.85) 0%	2.18 (1.22) 0%
2	42 (15)	58.1 (7.8)	30 (71)	3.98 (1.71) 31%	5.67 (1.26) 66%	5.45 (1.37) 56%	2.79 (0.95) 0%	3.70 (1.66) 16%
3	37 (14)	57.9 (7.4)	29 (78)	3.79 (1.83) 22%	4.13 (1.69) 17%	3.67 (1.52) 9%	4.75 (1.09) 26%	4.46 (1.65) 26%
4	171 (62)	56.6 (8.0)	152 (89)	4.68 (1.99) 41%	6.30 (1.13) 82%	6.31 (1.01) 83%	6.49 (0.70) 91%	6.22 (1.18) 82%

Note. In this table, we report the mean score of the RAPA-1 scale and the percentage of patients who met recommended guidelines, corresponding to a score of 6 or 7, at each time point.

**Table 5:** Multiple linear impacts of sociodemographic and clinical variables and SOC on the probability of belonging to the target physical activity profile (*n* = 272). Data are the *t*-test value (*t*) and the corresponding *p* value, the standardized regression coefficients (standardized β), and confidence interval (95%).

				95% Confidence Interval	
Predictor	*t*	*p*	Standardized β	Lower	Upper
Age	−1.32	0.187	−0.08	−0.20	0.04
Gender (male–female)	2.14	0.033	0.35	0.03	0.67
Obesity (no–yes)	2.11	0.036	0.34	0.02	0.66
SOC	3.19	0.002	0.19	0.07	0.31

**Table 6:** Multiple linear impacts of the probability of belonging to the target physical activity profile and SOC on HRQoL at the final follow-up (*n* = 170). Data are the *t*-test value (*t*) and the corresponding *p* value, the standardized regression coefficients (standardized β), and confidence interval (95%).

				95% Confidence Interval	
Predictor	*t*	*p*	Standardized β	Lower	Upper
Prob. of class 4	2.17	0.031	0.16	0.01	0.31
SOC	3.67	<0.001	0.27	0.13	0.3

## Data Availability

The data presented in this study are available on request from the corresponding author. The data are not publicly available, due to privacy and ethical restrictions.
